# Photoprotective Effects of Cannabidiol against Ultraviolet-B-Induced DNA Damage and Autophagy in Human Keratinocyte Cells and Mouse Skin Tissue

**DOI:** 10.3390/molecules27196740

**Published:** 2022-10-10

**Authors:** Yanmei Li, Dan Hao, Danfeng Wei, Yue Xiao, Lian Liu, Xiaoxue Li, Lian Wang, Yu Gan, Wei Yan, Bowen Ke, Xian Jiang

**Affiliations:** 1 Department of Dermatology, West China Hospital, Sichuan University, Chengdu 610041, China; 2 Laboratory of Dermatology, Clinical Institute of Inflammation and Immunology (CIII), Frontiers Science Center for Disease-Related Molecular Network, West China Hospital, Sichuan University, Chengdu 610041, China; 3 Department of Anesthesiology, Laboratory of Anesthesia and Critical Care Medicine, National-Local Joint Engineering Research Centre of Translational Medicine of Anesthesiology, West China Hospital, Sichuan University, Chengdu 610041, China

**Keywords:** autophagy, cannabidiol, DNA damage, oxidative stress, photodamage

## Abstract

Cannabidiol (CBD) has emerged as a phytocannabinoid with various beneficial effects for the skin, including anti-photoaging effects, but its mechanisms of action are not fully elucidated. The study assessed CBD’s photoprotective effects against acute ultraviolet B (UVB)-induced damage in HaCaT human keratinocyte cells and murine skin tissue. CBD (8 μM) alleviated UVB-induced cytotoxicity, apoptosis, and G2/M cell cycle arrest in HaCaT cells. The contents of γH2AX and cyclobutane pyrimidine dimers were decreased after CBD treatment. CBD reduced the production of reactive oxygen species and modulated the expression of antioxidant-related proteins such as nuclear factor erythroid 2-related factor 2 in UVB-stimulated HaCaT cells. Furthermore, CBD mitigated the UVB-induced cytotoxicity by activating autophagy. In addition, a cream containing 5% CBD showed effectiveness against UVB-induced photodamage in a murine model. The CBD cream improved the skin’s condition by lowering the photodamage scores, reducing abnormal skin proliferation, and decreasing expression of the inflammation-related protein cyclooxygenase-2 in UVB-irradiated skin tissue. These findings indicate that CBD might be beneficial in alleviating UVB-induced skin damage in humans. The photoprotective effects of CBD might be attributed to its modulatory effects on redox homeostasis and autophagy.

## 1. Introduction

A surging body of evidence supports the therapeutic potential of cannabidiol (CBD) for various skin conditions, including acne, eczema, dermatitis, and skin barrier dysfunction [1]. Certain preclinical studies have revealed that CBD can exert protective effects against ultraviolet (UV)-induced skin damage. For instance, in vitro studies using 2D- and 3D-cultured skin cell models showed that CBD protects human skin keratinocyte cells from UV irradiation by alleviating cellular oxidative stress [2]. In addition, CBD reduces ultraviolet B (UVB)-induced apoptotic pathways by modulating cell death-related proteins such as pro-apoptotic prostaglandin, p-p38, and caspase-8 [3]. CBD has also been reported to restore redox balance by mediating UVB-induced lipid peroxidation products [4]. Furthermore, CBD counteracts UV-induced lipid modification and protein metabolism in rat skin tissue by increasing the transcriptional activity of nuclear factor erythroid 2-related factor 2 (*Nrf2*) and reducing the levels of pro-inflammatory factors such as nuclear factor kappa B (NF-κB) and tumor necrosis factor α (TNF-α) [5]. Proteomic studies have revealed that several proteins are involved in the antioxidant effects of CBD in skin cells and tissues [6].

Redox homeostasis-related proteins, such as the transcription factor *Nrf2*, play a critical role in the cellular antioxidant defense against UVB-induced photodamage in skin cells and tissues. Compared with wild-type mice, *Nrf2*-knockout mice have severer and longer lasting sunburn on their ear skin after UVB irradiation [7]. Conversely, skin-derived precursor cells with an overexpressed *Nrf2* gene are more resistant to UV damage in 3D skin culture models, whereas *Nrf2*-silenced skin precursor cells are more vulnerable to UVB irradiation [8]. In addition to *Nrf2*, autophagy (also referred to as macroautophagy) also provides an important endogenous defense mechanism against oxidative stress by mediating skin homeostasis [9]. Autophagy participates in numerous cellular events, including the regulation of DNA damage and repair, programmed cell death, cell aging, and cytokine secretion [10]. Our group has demonstrated that natural products such as sanshool can protect skin cells against UVB-induced skin damage via the induction of autophagy [11]. Notably, specific autophagy markers—namely, ubiquitin-binding protein p62 (P62)—can bind to microtubule-associated protein 1A/1B-light chain 3 (LC3) to facilitate the process of autophagy [12]. Although CBD has been reported to modulate the biosynthesis, degradation, and expression of proteins involved in cellular apoptosis, inflammation, and redox balance in keratinocytes exposed to UVB irradiation, its anti-photodamage effects—especially its effects on DNA damage—have not been fully elucidated. Whether or not CBD protects cells against photodamage via autophagy is also not clear. In this study, we aimed to explore the effects and mechanisms of CBD against acute UVB-induced skin damage by evaluating: (1) the effects of CBD on UVB-induced cytotoxicity, apoptosis, cell cycle arrest, and DNA damage in HaCaT keratinocyte cells; (2) CBD’s antioxidant and pro-autophagy effects; and (3) the efficacy of a CBD cream’s photoprotection against UVB irradiation in a murine model.

## 2. Results

### 2.1. CBD Alleviates UVB-Induced Cytotoxicity and Apoptosis in HaCaT Keratinocyte Cells

**Figure 1:**
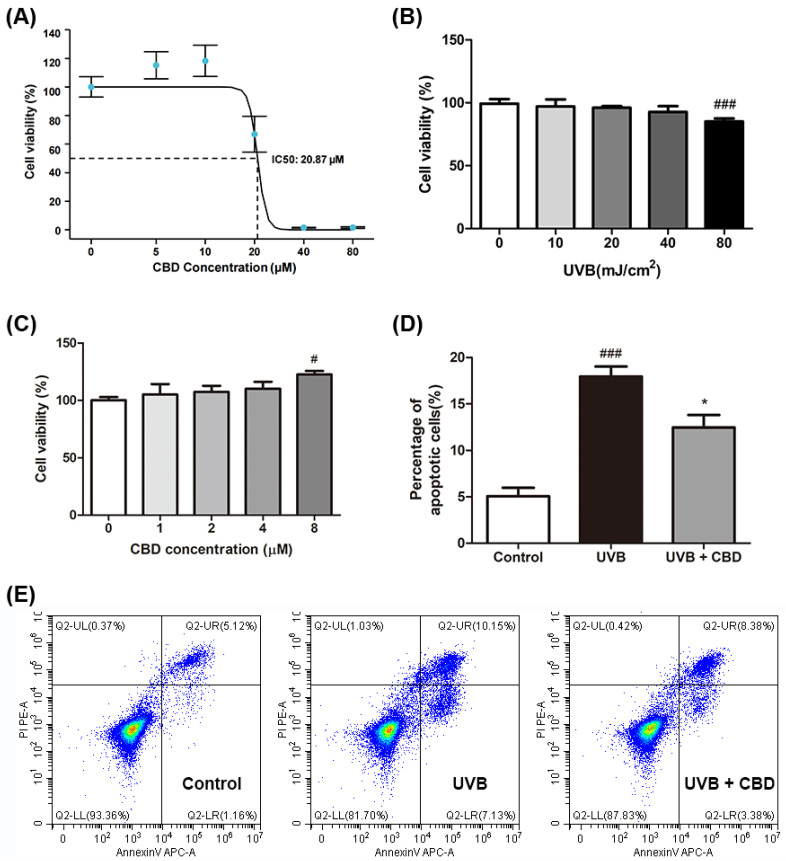
Effects of CBD on UVB-induced cytotoxicity and apoptosis in HaCaT cells: (**A**) Cells were incubated with CBD (5, 10, 20, 40, and 80 μM) for 24 h, and the cell proliferation activity of each group was detected by the CCK-8 assay (*n* = 6). (**B**) To detect the effects of UVB irradiation on cell viability, HaCaT cells were irradiated with UVB at varying doses and cultured for 24 h (*n* = 6). (**C**) Effect of CBD treatment for 24 h on the viability of UVB-irradiated HaCaT cells. Cell viability was measured by the MTT method. HaCaT cells were irradiated at 80 mJ/cm^2^, followed by incubation with CBD (0, 1, 2, 4, and 8 μM) for 24 h (*n* = 6). (**D**,**E**) HaCaT cells were irradiated at 80 mJ/cm^2^, followed by incubation with 8 μM CBD for 24 h. Cells were harvested and stained with Annexin V/PI staining reagents to measure the population of apoptotic cells using a flow cytometric assay. Results are presented as the mean ± SD (*n* = 3); ^#^ *p* < 0.05 and ^###^ *p* < 0.001 as compared with the control group; * *p* < 0.05 as compared with the UVB-irradiated group.

The cell viability experiment for HaCaT cells using a CCK-8 kit showed that the IC_50_ of CBD was 20.87 µM, and CBD treatment improved the viability of UVB-irradiated cells at a concentration of 10 μM (Figure 1A). UVB (40 and 80 mJ/cm^2^; 24 h) induced cytotoxicity and suppressed the viability of HaCaT cells to 93.4% and 84.7%, respectively (Figure 1B). A significant inhibition was observed in cells exposed to UVB of 80 mJ/cm^2^, so the UVB radiation intensity of 80 mJ/cm^2^ was selected as the model group for further experiments. Compared to the model group, treatment with CBD (at concentrations of 1, 2, 4, and 8 μM) restored the cell viability in a dose-dependent manner, by 5.0%, 7.3%, 10.2%, and 22.6% (*p* < 0.05), respectively (Figure 1C). This cytoprotective effect was further supported by data from the apoptotic assay. UVB irradiation increased the population of apoptotic cells (2.6-fold) compared to control group. CBD (8 μM) alleviated UVB-induced photodamage by reducing the rate of apoptosis by 30.6% (Figure 1D,E).

### 2.2. CBD Regulates Cell Cycle Progression and Reduces UVB-Induced DNA Damage in HaCaT Cells

**Figure 2:**
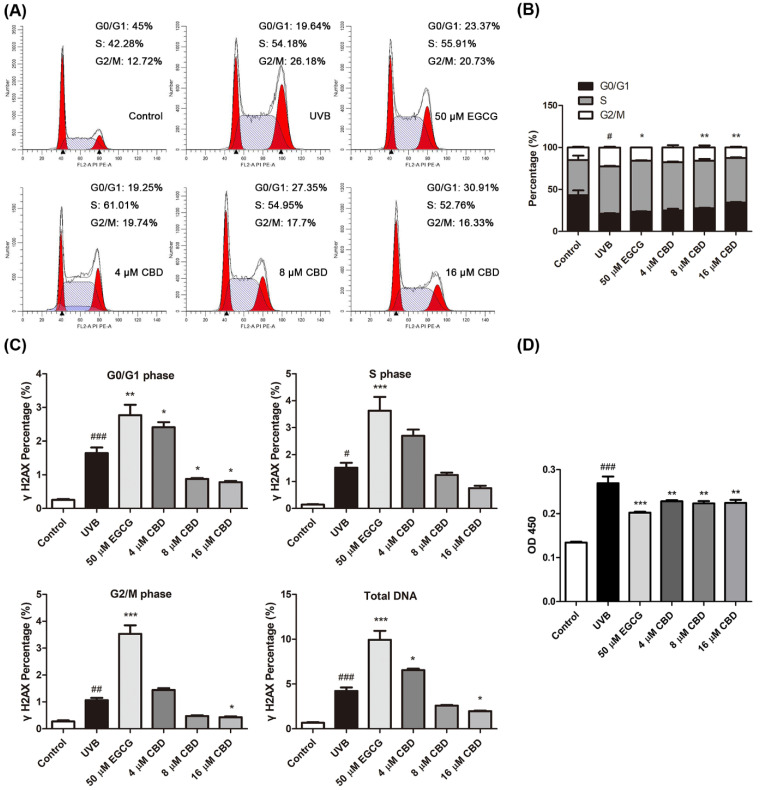
Effects of CBD on the cell cycle progression and DNA damage in photodamaged HaCaT cells. Cells were irradiated with UVB (80 mJ/cm^2^), followed by incubation with EGCG (50 μM) or CBD (4, 8, or 16 μM) for 24 h. (**A**,**B**) Cell suspensions from the control, UVB, UVB + EGCG, and UVB + CBD groups were stained with PI/RNase reagent and analyzed by flow cytometric assay for the cell cycle stages. (**C**) Cells were permeabilized and intracellularly stained with γH2AX and PI/RNase reagent to detect γH2AX. (**D**) Cell DNA was extracted and the CPD content was determined via ELISA using a CPD antibody. Data were obtained from three independent experiments; ^#^ *p* < 0.05, ^##^ *p* < 0.01, and ^###^ *p* < 0.001 as compared with the control group; * *p* < 0.05, ** *p* < 0.01, and *** *p* < 0.001 as compared with the UVB-irradiated group.

Epigallocatechin gallate (EGCG)—a polyphenol with reported photoprotective effects against UVB-induced photodamage [13]—was used as a positive control to study the photoprotective effects of CBD in keratinocytes. Flow cytometry analysis showed that the exposure to UVB (80 mJ/cm^2^) disrupted the cell cycle distribution in HaCaT cells and increased the G2/M phase by 51.9% compared to the control group, in which the cell cycle distribution was mainly in the G0/G1 phase. Treatments with CBD (4, 8, and 16 μM) or EGCG (50 μM) had comparable mitigative effects on the UVB-induced cell cycle shift, as they decreased the G2/M phase (by 22.6%, 29.9%, 45.2%, and 30.6%, respectively) and increased the G0/G1 phase (by 17.4%, 29.9%, 60.9%, and 10.5%, respectively) (Figure 2A,B). In addition, the DNA-protective effect of CBD was evaluated by measuring the levels of two DNA damage-related biomarkers—namely, phosphorylated histone H2AX (γH2AX) and cyclobutane pyrimidine dimers (CPDs)—in HaCaT cells. The flow cytometry analysis showed that the level of γH2AX—an indicator of DNA double-strand breaks (DSBs) [14]—in the total DNA content was significantly higher (5.3-fold) in the UVB-treated group than in the control group. Compared to the UVB model group, treatment with CBD (8 and 16 μM) reduced the total intracellular γH2AX content by 38.4% and 53.4%, respectively (Figure 2C). The most prevalent products of DNA produced by direct UVB radiation are cyclobutane pyrimidine dimers (CPDs), resulting in C-to-T mutations [15]. Data obtained from the enzyme-linked immunosorbent assay (ELISA) assay showed that the CPD content in the UVB model group was one-fold higher than that in the control group. Treatment with CBD (4, 8, and 16 μM) and EGCG (50 μM) reduced the CPD contents in UVB-treated cells by 15.2%, 17.0%, 16.6%, and 24.9%, respectively (Figure 2D).

### 2.3. CBD Reduces UVB-Induced Oxidative Stress in HaCaT Cells

**Figure 3:**
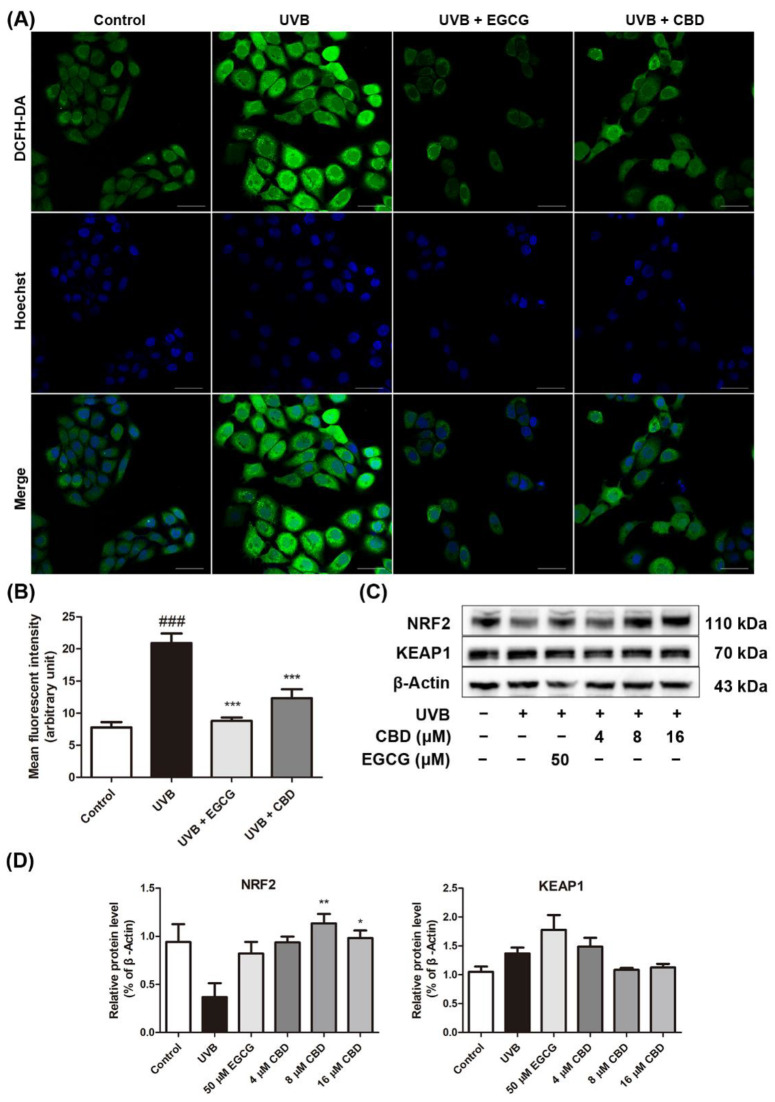
Effects of CBD on the oxidative stress in UVB-irradiated HaCaT cells: HaCaT cells were irradiated at 80 mJ/cm^2^, followed by incubation with EGCG (50 μM) or CBD (8 μM) for 24 h. (**A**) Cells were incubated with a fluorescent probe (DCF-DA) for qualitative analysis using a confocal microscope, and (**B**) the mean fluorescence intensity (MFI) was measured using ImageJ software. (**C**,**D**) Cells were irradiated with UVB (80mJ/cm^2^), followed by incubation with EGCG (50 μM) or CBD (4, 8, or 16 μM) for 24 h. Expression levels of NRF2 and KEAP1 were determined by Western blot analysis. Scale bar = 50 μm. Results are presented as the mean ± SD (*n* = 3); ^###^ *p* < 0.001 as compared with the control group; * *p* < 0.05, ** *p* < 0.01, and *** *p* < 0.001 as compared with the UVB-irradiated group.

A fluorescent probe (DCF-DA) was used to measure the production of total ROS in HaCaT cells. Data from the immunofluorescent assay showed that the ROS levels in HaCaT cells after UVB irradiation were increased (1.7-fold) as compared to those in the control group. The generation of ROS was elevated in the UVB model group, as reflected by the increased brightness and number of green fluorescent spots. The ROS levels were reduced after the treatment with CBD (8 μM) or EGCG (50 μM) (Figure 3A). Furthermore, the mean fluorescence intensity (MFI) was obtained as a quantitative index for ROS production. The MFI of the UVB + CBD group and the UVB + EGCG group was significantly lower than that of the UVB model group (by 41.0% and 57.9%, respectively) (Figure 3B). Western blot analysis showed that UVB irradiation decreased the protein expression level of NRF2 (by 61.0%) and increased KEAP1 expression (by 30.3%) as compared to the control group. This effect was counteracted by the treatment with CBD (8 and 16 μM), which increased the expression level of NRF2 (by 2.1-fold and 1.7-fold, respectively) and downregulated the levelof KEAP1 (by 20.7% and 17.8%, respectively) (Figure 3C,D).

### 2.4. CBD Activates Autophagy in HaCaT Cells

**Figure 4:**
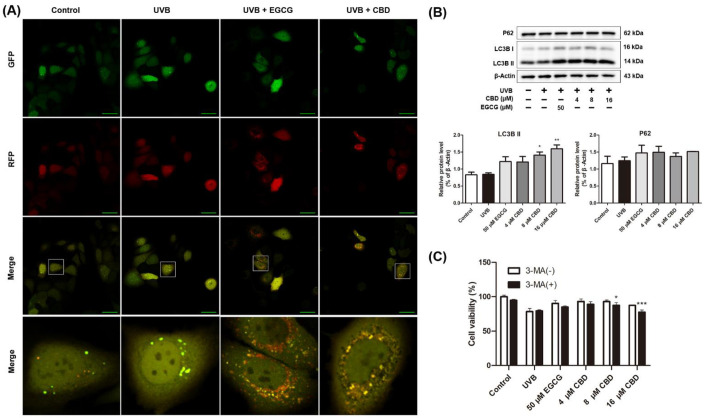
Effects of CBD on autophagy in UVB-irradiated HaCaT cells: (**A**) Cells were irradiated with UVB (80 mJ/cm^2^), followed by incubation with the mRFP–GFP–LC3 adenovirus and EGCG (50 μM) or CBD (8 μM) for 24 h. LC3 staining was observed using a confocal microscope. (**B**) Cells were irradiated with UVB (80 mJ/cm^2^), followed by incubation with EGCG (50 μM) or CBD (4, 8, or 16 μM) for 24 h. Expression levels of P62 and LC3B were determined by Western blot analysis. (**C**) Cell proliferation activity of each group pretreated with or without 3-MA was detected by the MTT method. Scale bar = 50 μm. Results are presented as the mean ± SD (*n* = 3); * *p* < 0.05, ** *p* < 0.01, and *** *p* < 0.001 as compared with the UVB-irradiated group.

We detected autophagy by tracing the formation and degradation of autophagosomes via the fusion of LC3. The tandem monomeric red fluorescent protein (mRFP)–green fluorescent protein (GFP)–LC3 adenovirus was constructed to monitor the induction of autophagy [12]. After the mRFP–GFP–LC3 adenovirus was introduced, GFP- (green dots) and mRFP-stained (red dots) signals were observed in the control and UVB groups. The number of GFP and mRFP signals increased in the UVB + CBD and UVB + EGCG groups, and a large number of orange–yellow spots were observed in the merged images, suggesting the presence of autophagosomes (Figure 4A). Next, the expression levels of LC3B I/II and p62 were measured. Compared with the control group, LC3B I/II and P62 in the UVB group had no significant changes. Compared with the UVB group, treatment with CBD (8 and 16 μM) upregulated the expression of LC3B II (by 66.6% and 88.6%, respectively). Interestingly, no significant changes in the expression of P62 were observed (Figure 4B). HaCaT cells were pretreated with the autophagy inhibitor 3-methyladenine (3-MA) to further verify CBD’s pro-autophagy effect. The results showed that the cell proliferation activity of CBD (8 and 16 μM) pretreated with 3-MA was lower than that in the groups without treatment with 3-MA (by 6% and 11.3%, respectively) (Figure 4C).

### 2.5. CBD Exerted a Photoprotective Effect against UVB-Induced Skin Damage in a Murine Model

**Figure 5:**
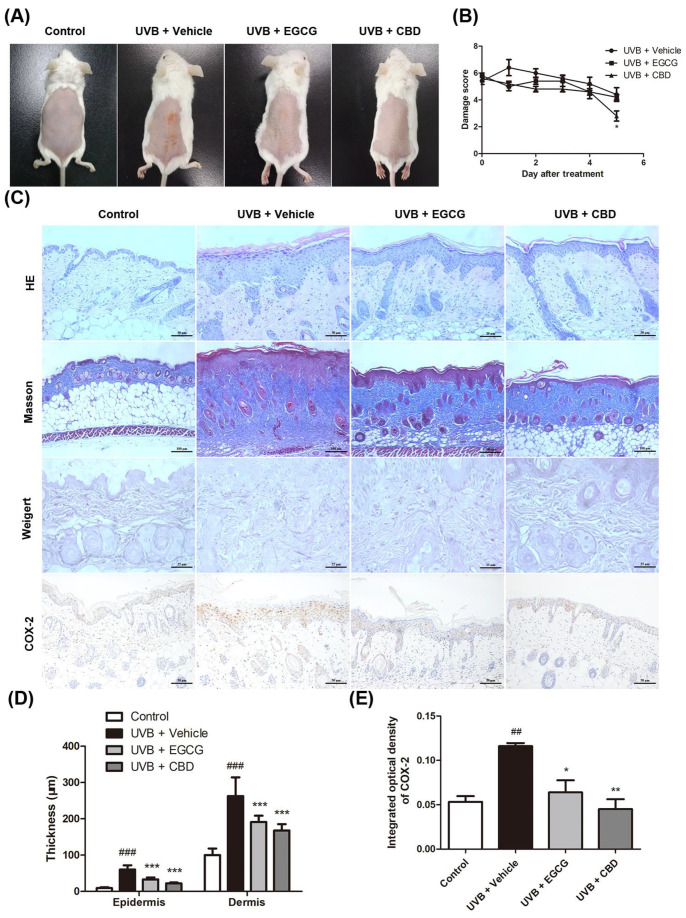
Protective effects of cannabidiol against skin photodamage in murine models: The backs of BALB/c mice were irradiated with UVB (120 mJ/cm^2^) once per day for 5 consecutive days, and then CBD or the vehicle was topically applied once per day for 5 days. The groups of mice were either (i) unexposed (control), (ii) exposed to UVB radiation and treated topically with CBD (0.1 mg/cm^2^) (UVB + CBD), (iii) exposed to UVB and treated with an equal amount of the vehicle (UVB + vehicle), or (iv) exposed to UVB radiation and treated topically with EGCG (0.75 mg/cm^2^) (UVB + EGCG). Skin biopsies were processed for staining 24 h after the last treatment. (**A**) Representative features of the dorsal skin 24 h after the last treatment. (**B**) Photodamage scores were obtained before the first treatment and 24 h after each treatment. (**C**) Hematoxylin and eosin (HE), Masson, and Weigert’s elastic-fiber staining of skin samples, along with immunohistochemical staining for COX-2, were performed. (**D**) The thickness of the epidermal and dermal layers and (**E**) the integrated optical density of COX-2 expressed as positive area % were measured using ImageJ software. Data are presented as the mean ± SD (*n* = 5); ^##^ *p* < 0.01, ^###^ *p* < 0.001 as compared with the control group; * *p* < 0.05, ** *p* < 0.01, and *** *p* < 0.001 as compared with the UVB-irradiated group.

A single dose of UVB irradiation (120 mJ/cm^2^) per day for five consecutive days on murine dorsal skin was used to establish an animal model of UVB-induced photodamage. The model group (UVB + vehicle) developed erythema on the back skin, and the size of wrinkles and scaling after UVB exposure gradually increased. In the UVB + CBD group, the area of skin with erythema and scales decreased after UVB exposure without the formation of large transverse wrinkles (Figure 5A). The daily damage score showed that the UVB + CBD group had lower skin damage severity after UVB exposure (at day 6), as compared with that in the model group (Figure 5B). Histological results showed that the epidermal thickening was less in the CBD treatment group than in the model group, and the number of collagen fiber bundles in the model group was higher than that in the CBD treatment group. The elastic fibers in the model group were broken, thickened, and twisted; conversely, the elastic fibers were uniform and neat in the CBD treatment group. We also examined the expression of COX-2 in the murine skin tissue. The results of IHC showed that COX-2 was highly expressed in the epidermis and the cytoplasm in the model group, whilst it was weakly detected in the control and CBD treatment groups (Figure 5C). The histopathological changes showed that the epidermis and dermis were thinner in the UVB + CBD and UVB + EGCG groups than in the model group (*p* < 0.01), and the COX-2-positive staining area was reduced in the UVB + CBD and UVB + EGCG treatment groups compared with the model group (*p* < 0.01) (Figure 5D,E).

## 3. Discussion

Excessive exposure to UVB leads to cellular oxidative damage and various forms of cell death, including apoptosis and necrosis. Here, our data suggest that CBD ameliorated the UVB-induced cytotoxicity by enhancing cell proliferation and reducing cell apoptosis. These results are consistent with the findings of previous studies. For example, a study showed that CBD can reduce the apoptosis of UV-irradiated skin fibroblasts [16]. One of the hallmark events of UVB irradiation is the induction of cellular DNA damage. DNA damage can cause cell cycle arrest in the G1 or G2 phase, or it can delay replication in the S phase. As a counteractive mechanism, cells can initiate several responsive pathways to activate DNA repair and cell cycle arrest after UVB irradiation [17]. DNA damage response (DDR) is a signal transduction pathway driven by protein phosphorylation. It is generally believed that three PI3K-related kinase (PIKK) family kinases play a leading role in DDR: DNA-dependent protein kinase (DNA-PK), ataxia telangiectasia mutant kinase (ATM), and Rad3-related kinase (ATR). Impairing ATR signaling to Chk1 results in G2/M arrest [18]. Ultraviolet radiation induces DNA double-strand breaks (DSBs) in cells, activates ATM or ATR, and rapidly phosphorylates H2AX to form γH2AX, promoting DNA damage-repair events in the cells [19]. CPDs are the main photochemical products generated by direct damage to DNA caused by UVB irradiation, and they are a principal mediator of the cellular transcriptional response; they promote the accumulation of γH2AX and DSBs [20]. Our study showed that cells were arrested in the G2/M phase after UVB irradiation, while CBD alleviated cell cycle arrest. To the best of our knowledge, this is the first report showing that CBD decreases the contents of γH2AX and CPDs and mitigates UVB-induced cell cycle arrest in HaCaT cells. Interestingly, we found that higher concentrations of CBD (8 or 16 μM) can reduce γH2AX, but a lower concentration of CBD (4 μM) increased γH2AX; the mechanism is unclear. In the future, we will continue to study the effects of CBD on cell cycle checkpoints and DNA damage-repair pathways.

Excessive generation of ROS can disrupt cellular redox homeostasis and exacerbate DNA damage, leading to cell apoptosis and inflammation [21,22]. Antioxidant capacity is also an important indicator for evaluating photoprotective capacity. In vitro studies found that CBD exerts antioxidant effects via electron donation mechanisms [23]. Studies have demonstrated that NRF2 and its main negative regulator KEAP1 play important roles in redox homeostasis, inflammation, and metabolic regulation [24]. Our results are consistent with previous reports showing that CBD significantly reduced the production of ROS and activated the activity of antioxidant-related enzymes [25].

Furthermore, we found that CBD mitigated the UVB-induced cytotoxicity by activating autophagy. Autophagy is considered to be a double-edged sword for cell survival and cell death. Some studies have suggested that CBD activates autophagy to regulate cell death events. For example, CBD induced the death of melanoma cells by initiating autophagy [26]. However, CBD inhibited NF-κB and induced autophagy to protect SH-SY5Y cells from mitochondrial dysfunction [27]. Under stress conditions, such as oxidative stress, autophagy usually plays a protective role to maintain cell homeostasis [28]. Moreover, autophagy is involved in the repair of DNA damage caused by UV. The most important mechanism for protecting DNA from UV damage is nucleotide excision repair (NER). Studies have reported that autophagy positively regulates NER by enhancing the recognition of DNA damage by the DNA damage-sensor proteins XPC and DDB2 [29]. In addition, studies have found that the UV radiation resistance-associated gene (UVRAG) may act as a signal transduction center that simultaneously activates DNA damage repair and autophagy [30]. P62 is an autophagy substrate, and its expression was downregulated with the activation of autophagy. Interestingly, we found that P62 was not significantly decreased after CBD treatment. This may be due to CBD-induced high expression of NRF2, which is a transcription factor for P62 [31]. Combined with evidence reported from other studies, we speculate that CBD acts as an autophagy agonist to improve cellular resistance to UVB irradiation.

A short period of exposure to excessive UV (e.g., sunburn) can cause acute skin photodamage. Physiological characteristics of acute photodamage include erythema, edema, pain, blisters, and scales. The histopathological manifestations can be observed as epidermal hyperplasia, thickened stratum corneum, sunburn cells, dermal inflammation infiltration, collagen and elastic fiber damage, and vasodilation [32]. In this study, we found that the topical use of CBD improved the appearance and histological changes of photodamaged murine skin. Furthermore, CBD reduced the levels of COX-2 in murine skin tissue. COX-2 is considered to be one of the early markers of UVB exposure [33]. COX-2 expression increased rapidly after UVB exposure, accompanied by the elevated release of chemokines and cytokines, as a response to skin damage [34]. Studies have found that increased COX-2 expression may be due to the generation of ROS and phosphorylation of epidermal growth factor receptors caused by UVB exposure, leading to the activation of COX-2 transcription factors [35]. Previous studies also reported that ROS contributed to the structural degradation of intact fibers and the promotion of abnormal fibrin deposition [36]. We speculate that the protective effects of CBD on epidermal and dermal fibers may be related to the amelioration of oxidative stress and inflammation induced by UVB.

## 4. Materials and Methods

### 4.1. Chemical and Reagents

CBD was provided by Yunnan Hempmon Pharmaceutical Co., Ltd. (Kunming, China; purity of ≥98%). A stock solution of CBD (16 mM) was prepared in dimethyl sulfoxide (DMSO; Sigma-Aldrich, St. Louis, MO, USA) and diluted with Dulbecco’s modified Eagle medium (DMEM; Gibco, Grand Island, NY, USA) to reach the desired final concentration with less than 0.1% DMSO. A CBD cream (5%) was prepared by mixing the CBD isolate and a vehicle base cream (provided by Yunnan Hempmon Pharmaceutical Co., Ltd., Kunming, China). The vehicle cream contained ingredients including water, disodium ethylenediaminetetraacetic acid (EDTA), p-anisic acid, cetearyl glucoside, sorbitan caprylate, cetyl alcohol, stearyl alcohol, dioctyl carbonate, shea butter, ammonium acryloyldimethyltaurate/VP copolymer, glycerin, phenoxyethanol, and sodium hydroxide. Epigallocatechin gallate (EGCG) (purity ≥ 98%) was purchased from Chemleader Biomedical (Shanghai, China). The 3-Methyladenine (3-MA) was purchased from Selleck. The 3-(4,5-Dimethylthiazol-2-yl)-2,5-diphenyltetrazolium bromide (MTT) and 2′,7-dichlorodihydrofluorescein-diacetate (DCFH-DA) were purchased from Sigma-Aldrich.

### 4.2. Cell Culture, UVB Irradiation, and Treatments

Human-immortalized keratinocytes (HaCaT cells) were obtained from the Cell Bank of Academia Sinica (Shanghai, China). The UVB irradiation was performed with a SH4 UV phototherapy instrument (Sigma, Shanghai, China) with a peak irradiance of 313 nm. The UVB radiation intensity was measured with a UVB radiometer (Photoelectric Instrument, Beijing Normal University, Beijing, China). The UVB radiation intensity at the fixed position measured by the UV radiometer was 0.825 mW/cm^2^; UVB dose (mJ/cm^2^) = UVB radiation intensity (mW/cm^2^) × time (s). Therefore, the irradiation time required for an 80 mJ/cm^2^ dose of UVB is 96 s. The cells were covered with a thin layer of PBS during UVB exposure. After UVB irradiation, the PBS was replaced with fresh media containing 0.1% DMSO or different concentrations of treatments, while in the sham irradiation group, fresh medium containing 0.1% DMSO was added and incubated for 24 h.

### 4.3. Cell Viability Assays and Apoptosis Detection

Cell viability assays were performed using MTT or cell counting kit-8 (CCK-8; Dojindo, Kumamoto, Japan) reagents according to the manufacturer’s protocols. The cell viability was determined colorimetrically. The apoptosis assay was performed by using an Annexin V-FITC/PI apoptosis kit (KeyGen Biotech, Nanjing, China). The cells were then stained with 5 μL of Annexin V solution and 1 μL of propidium iodide (PI) working solution before being analyzed using a flow cytometer (Beckman CytoFLEX, Brea, CA, USA).

For the autophagy inhibition assay, HaCaT cells were pretreated with 3-MA solution at a final concentration of 5 mmol/L and incubated for 6 h. Then, the cells were treated according to their groups. Cell viability assays were performed using MTT.

### 4.4. Detection of Cell Cycle and γH2AX

HaCaT cells were divided into control, UVB, UVB + EGCG (50 μM), and UVB + CBD (4, 8, and 16 μM) groups. After the UVB irradiation or sham irradiation, the cells were incubated with different treatments for 24 h. The cells were then collected, fixed, and permeabilized using 70% ethanol overnight at 4 °C. Subsequently, the cells were intracellularly stained with 2 μL of H2A.X Phospho (Ser139) FITC (BioLegend Technology, San Diego, CA, USA) for 1 h and then stained with 500 μL of PI/RNase staining solution (KeyGen Biotech, Nanjing, China) for 30 min. Finally, the cells were detected by flow cytometry.

### 4.5. Measurement of CPDs by ELISA

ELISA for CPDs was performed using murine monoclonal anti-CPDs (Cat. CAC-NMDND-001, Clone TDM-2, CosmoBio, Tokyo, Japan) following the manufacturer’s protocol (Cat. NMDND001). Briefly, DNA was isolated from cells using the DNeasy blood and tissue kit (QIAGEN, Hilden, Germany) and quantified with a NanoDrop spectrophotometer (Thermo Fisher Scientific, Waltham, MA, USA). Then, the DNA of each group was diluted to 0.2 µg/mL with PBS and denatured at 100 °C. After cooling, the samples were added (50 μL/well) to ELISA plates, with three replicate wells in each group. After being washed five times, the samples were incubated with 2% fetal calf serum, incubated with 1:1000 diluted CPD primary antibody, and then incubated with horseradish peroxidase (HRP)-labeled goat anti-mouse antibody. Next, the samples were incubated with 100 μL of substrate solution for 30 min, and the enzyme reaction was stopped with 100 µL of stop solution. Finally, the absorbance of each well was measured at 450 nm.

### 4.6. Measurement of ROS Levels

The intracellular accumulation of ROS was detected using DCFH-DA. HaCaT cells were irradiated with UVB or sham irradiation, followed by incubation. The cells were incubated with a 10 μM DCFH-DA probe solution and 10 μg/mL Hoechst33258 solution at 37 °C for 20 min. After washing, 3 mL of PBS was added to each well, and the production of ROS was observed using an A1R MP + multiphoton confocal microscope (Nikon, Tokyo, Japan). MFI was measured using the ImageJ software.

### 4.7. Immunocytochemistry to Detect Changes in Autophagy Flux

After UVB irradiation or sham irradiation, 0.5 μL of recombinant mRFP–GFP–LC3 adenovirus (Hanbio, Shanghai, China) was added to each well, and different concentrations of treatments were added at the same time according to the grouping. After incubation for 24 h, the LC3 expression was observed using a confocal microscope. Result judgment: mRFP (red fluorescent protein) and GFP (green fluorescent protein) jointly labeled LC3. The yellow spots that appeared after the merging of the red and green fluorescence indicated autophagosomes (early autophagy), and the red spots indicated autophagolysosomes (late autophagy). The strength of the autophagic flux was determined by observing the number of yellow and red spots [37].

### 4.8. Western Blot Assays

After being treated according to their groups, the cells were harvested and lysed. The supernatant was mixed with sodium dodecyl sulfate (SDS) sample buffer and boiled for 5 min. Equal amounts of protein were separated by SDS−polyacrylamide gel electrophoresis (SDS–PAGE), followed by semidry blotting on a polyvinylidene difluoride membrane (Thermo Fisher Scientific, Waltham, MA, USA). After blocking the membrane with 5% (*w*/*v*) Tris-buffered saline with Tween 20 (TBST) and non-fat dry milk, the primary antibodies were added. The secondary antibodies—HRP-conjugated anti-mouse/anti-rabbit IgG (GenScript, Nanjing, China)—were detected by enhanced chemiluminescence (ECL) using an ECL Western Blotting Substrate (Bio-Rad, Hercules, CA, USA). The antibodies used were as follows: LC3B (CST, Beverly, MA, USA); p62 (Proteintech, Wuhan, China); KEAP1 (Proteintech, Wuhan, China); NRF2 (Proteintech, Wuhan, China); and β-actin (Abbkine, Wuhan, China). The band densities were analyzed using the software ImageJ.

### 4.9. Animal Study

The animal experiments were approved by the Biomedical Research Ethics Committee at West China Hospital (2019186A). The dorsal skin of mice in the UVB-irradiated groups was exposed to UVB radiation at 120 mJ/cm^2^ once per day for five consecutive days. This radiation dose was selected based on our pre-experimental studies (see the Supplementary Materials). The UVB source was an SH4 UV phototherapy instrument. The fixed irradiation distance was ~28 cm, and the radiation intensity was 0.25 mW/cm^2^. Therefore, the irradiation time required for a 120 mJ/cm^2^ dose of UVB was 8 min. Intervention was performed after the entire model building was completed. The groups of mice were as follows: (i) unexposed (control); (ii) exposed to UVB radiation and treated topically with CBD (0.1 mg/cm^2^) (UVB + CBD); (iii) exposed to UVB and treated with an equal amount of the vehicle (UVB + vehicle); and (iv) exposed to UVB radiation and treated topically with EGCG (0.75 mg/cm^2^) (UVB + EGCG) [38]. The degree of dorsal skin damage characteristics—including scarring/dryness, edema, erythema/hemorrhage, and excoriation/erosion—was evaluated based on the clinical skin severity score (Supplementary Materials: Table S1). Photodamage scores were obtained 24 h after each treatment. The mice were euthanatized 24 h after the last treatment. The dorsal skin was collected and embedded in paraffin for subsequent histological analysis.

### 4.10. Histology and Immunohistochemistry

The paraffin blocks were cut, mounted on glass slides, deparaffinized, and rehydrated with graded ethanol. The sections were stained with H&E, Masson, and Weigert’s elastic-fiber stain. COX-2 immunohistochemistry analysis was also performed. Briefly, the sections were incubated with diluted COX-2 (CST, Beverly, MA, USA) antibody solution (1:800), followed by incubation with a diluted secondary antibody (1:200). The epidermal and dermal thickness and staining intensity were quantified using ImageJ software [11]. In each section, 5 different sites of epidermis and dermis were selected randomly. For immunohistochemistry (IHC), the COX-2-positive area was counted in five high-power fields per section for each mouse.

### 4.11. Statistical Analyses

All the values are presented as the mean ± standard deviation (SD). Statistical analyses were mostly carried out using GraphPad Prism 5. Data were expressed as the mean of at least three independent experiments with multiple technical replicates in order to be eligible for the indicated statistical analyses. A one-way analysis of variance (ANOVA) with the Tukey–Kramer test or Dunnett’s test was used for most of the multiple comparisons. A logarithmic logistic model was used to analyze IC_50_ using R 3.6.3 software [39]. A *p*-value < 0.05 indicated a statistically significant difference.

## 5. Conclusions

The present study investigated the protective effects of CBD against UVB-induced photodamage. CBD protected HaCaT cells from the stimulation of UVB by promoting cell proliferation, alleviating apoptosis, releasing cell cycle arrest, and reducing the DNA damage. CBD also exerted cytoprotective effects by activating autophagy and reducing oxidative stress. In addition, topical application of CBD to the skin of UVB-irradiated mice lowered their photodamage scores, reduced abnormal skin proliferation, and decreased COX-2 expression in skin tissue. These findings, along with evidence reported from other studies, suggest that CBD is a phytocannabinoid with promising beneficial effects for the skin against UV-induced photodamage.

## Data Availability

Data are available upon request.

## Supplementary Materials

The following supporting information can be downloaded at: https://www.mdpi.com/article/10.3390/molecules27196740/s1, Figure S1: Skin appearance after different doses of UVB radiation; Figure S2: Histopathological changes in the skin of mice irradiated with 120 mJ/cm^2^ UVB; Table S1: List of photodamage scores.
