# Supplementary material for: Photoprotective Effects of Cannabidiol against Ultraviolet-B-Induced DNA Damage and Autophagy in Human Keratinocyte Cells and Mouse Skin Tissue
Source: Molecules. 2022 Oct 10;27(19):6740. doi: 10.3390/molecules27196740 (PMC9572435; doi:10.3390/molecules27196740)
Supplement: Supplementary file 1 [file molecules-27-06740-s001.zip › molecules-1948793-supplementary.pdf]

Supplementary Materials

# Photoprotective Effects of Cannabidiol against Ultraviolet-B-Induced DNA Damage and Autophagy in Human Keratinocyte Cells and Mouse Skin Tissue

**Yanmei Li [1,2], Dan Hao [1,2], Danfeng Wei [1,2], Yue Xiao [1,2], Lian Liu [1,2], Xiaoxue Li [1,2], Lian Wang [1,2], Yu Gan [3],**
**Wei Yan [1,2,*], Bowen Ke [3,*] and Xian Jiang [1,2,*]**

1 Department of Dermatology, West China Hospital, Sichuan University, Chengdu 610041, China
2 Laboratory of Dermatology, Clinical Institute of Inflammation and Immunology (CIII), Frontiers Science Center for Disease-Related Molecular Network, West China Hospital, Sichuan University, Chengdu 610041, China
3 Department of Anesthesiology, Laboratory of Anesthesia and Critical Care Medicine, National-Local Joint Engineering Research Centre of Translational Medicine of Anesthesiology, West China Hospital, Sichuan University, Chengdu 610041, China
* Correspondence: yanweihappyhappy@163.com (W.Y.); bowenke@scu.edu.cn (B.K.); jiangxian@scu.edu.cn (X.J.)

## Establishment of the animal model

The back of the mice was irradiated at different doses (90, 120, 180, 240, and 360 mJ/cm$^2$, n = 3) once a day. The dorsal skin was continuously irradiated until an obvious skin change, as the endpoint, was observed. Then images of lesions were taken (Figure S1). If the skin changes best matched the photodamage characteristics, HE staining was performed to detect the histopathological changes (Figure S2).

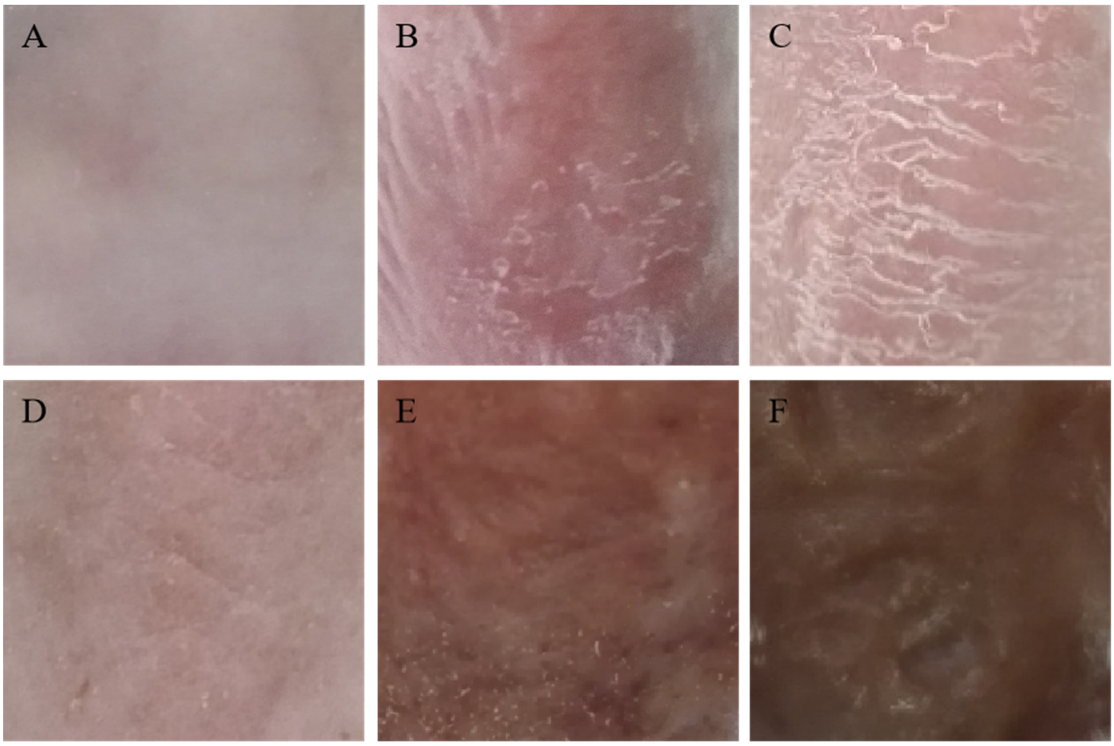


**Figure S1.** Different doses of UVB radiation caused skin photodamage of varying severity. (A)Normal mouse dorsal skin. Among the tested UVB doses, the skin changes caused by continuous exposure to UVB at doses of 90 mJ/cm$^2$ (B), 120 mJ/cm$^2$ (C) and 180 mJ/cm$^2$ (D) for 5 days were mainly erythema, scaly, and thickening. The skin changes caused by exposure at doses of 240 mJ/cm$^2$ (E) and 360mJ/cm$^2$ (F) for 3 days were mainly ulceration, bleeding, and crusting.

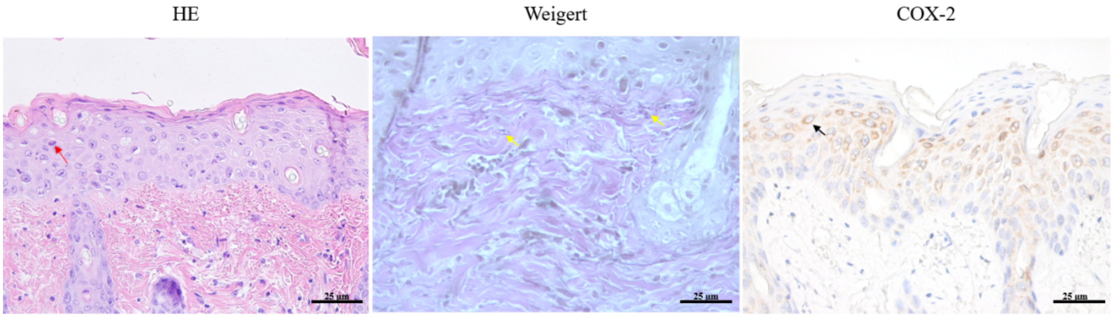


**Figure S2.** Histopathological results of mice irradiated with 120 mJ/cm$^2$ UVB for 5 days. After UVB irradiation, the stratum corneum was thickened and accompanied by cell edema, and apoptosis of some keratinocytes, namely "sunburn cells" (red arrow). In the dermis, inflammatory cells and collagen fibers were increased, the elastic fibers were arranged disorderly, broken, and twisted (yellow arrow). COX-2 immunohistochemical staining results showed COX-2 positive cells in the epidermis (black arrow).

## Photodamage scores

To our best knowledge, there is no unified evaluation standard for the skin photodamage condition. Photodamage score is generally graded according to severity. A scoring table was built based on the main skin change in this study (Table S1). Each mouse was evaluated for four indicators (erythema, thickness, scale, and texture) and scored according to the severity, with 0–2 points indicating none, mild, and severe respectively. The total score was calculated

for statistics.

**Table S1.** Photodamage scores

| Score | Erythema | Thickness | Scale | Texture |
|---|---|---|---|---|
| 0 | Pink | Soft | None | Smooth |
| 1 | Erythema | Thickened | Little | Rough |
| 2 | Ulcerate | Hypertrophic | Many | Wrinkled |
